# Real-time single-vessel analysis reveals vascular-type-dependent blood–brain barrier dysfunction in rodent models of status epilepticus and neuroinflammation

**DOI:** 10.1117/1.NPh.12.4.045004

**Published:** 2025-10-22

**Authors:** Bok-Man Kang, Juheon Lee, Sungjun Bae, Taeyoung Park, Seong-Eun Ryu, Yoonyi Jeong, Seung Won Chung, Hyun-Kyoung Lim, Kayoung Han, Minah Suh

**Affiliations:** a Sungkyunkwan University, Department of Biomedical Engineering (BME), Suwon, Republic of Korea; b IMNEWRUN Inc., Suwon, Republic of Korea; c Institute for Basic Science (IBS), Center for Neuroscience Imaging Research (CNIR), Suwon, Republic of Korea; d Sungkyunkwan University, Department of Intelligent Precision Healthcare Convergence (IPHC), Suwon, Republic of Korea; e Sungkyunkwan University, KIST-SKKU Brain Research Center, Suwon, Republic of Korea; f Sungkyunkwan University, Biomedical Institute for Convergence at SKKU (BICS), Suwon, Republic of Korea

**Keywords:** blood–brain barrier, vascular types, two-photon imaging, uniform manifold approximation and projection, pilocarpine-induced status epilepticus, lipopolysaccharide-induced neuroinflammation

## Abstract

**Significance:**

Recent evidence highlights significant heterogeneity of the blood–brain barrier (BBB) across vascular types, which becomes more pronounced under disease conditions. However, functional changes in vascular-type-dependent BBB leakage remain poorly characterized.

**Aim:**

We aimed to establish an analysis framework for quantifying single-vessel BBB total leakage and identifying vascular-type-dependent patterns of total leakage change in disease states.

**Approach:**

We introduce a method that combines *in vivo* real-time two-photon imaging with uniform manifold approximation and projection (UMAP)-based dimensionality reduction to assess BBB total leakage at the single-vessel level. Two rodent models were used, pilocarpine-induced status epilepticus (SE) and lipopolysaccharide-induced neuroinflammation (NI), which exhibit differential pathophysiological characteristics of BBB impairment.

**Results:**

Real-time imaging clearly showed arterial BBB leakage in SE, whereas leakage in NI was likely venous. Conventional intensity-based metrics, including area under the curve (AUC), intensity fold change ($\Delta F/F_{0}$), and averaged differential coefficient (ΔF/Δt), detected arteria-specific changes in SE but failed to capture vein-specific differences in NI. By contrast, UMAP-based analyses sensitively distinguished disease-specific total leakage patterns, allowing separation of SE from arterial data and NI from venous data.

**Conclusions:**

This integrated approach enables quantitative evaluation of vascular-type-dependent BBB total leakage and provides a platform for future studies on vessel-specific BBB alterations in neurological disorders.

## 1 Introduction

The blood–brain barrier (BBB) plays a central role in maintaining central nervous system (CNS) homeostasis by tightly regulating the exchange of substances between blood and brain parenchyma.^1^^–^^3^ Under physiological conditions, the BBB acts as a selective barrier that prevents circulating neurotoxins and immune cells from infiltrating the brain. Recent studies have indicated that the BBB is not a uniform structure. Instead, it displays heterogeneity across brain regions.^4^^,^^5^ Specifically, this heterogeneity in the BBB composition is highly dependent on the types of blood vessels, i.e., from arteries to capillaries to veins.^6^^,^^7^ Emerging research using single-cell RNA sequencing (scRNA-seq) has revealed distinct transcriptional patterns across arterial, capillary, and venous endothelial cells, indicating that each vascular type possesses unique functional characteristics.^8^ Under pathological conditions such as Alzheimer’s disease, these transcriptomic differences across vascular types are further amplified, with cell-type-specific dysregulation playing a key role in exacerbating BBB dysfunction.^9^^,^^10^ Given the functional specialization of each vascular segment, such molecular alterations are likely to manifest as vessel-type-specific changes in BBB permeability. This suggests that vascular-type-specific BBB dysfunction might drive heterogeneous permeability alterations during disease progression. Although previous studies have provided valuable molecular insights through gene and protein profiling, these approaches alone are insufficient to characterize the functional integrity of the BBB, particularly its permeability properties, which depend on disease stages. It remains unclear how such molecular profiles translate into actual changes in barrier function across vascular types. Therefore, direct and quantitative assessment of BBB leakage at the single-vessel level is essential for linking molecular alterations to functional outcomes. This approach is not only critical for understanding vessel-specific BBB disruption mechanisms but also essential for evaluating the efficacy of targeted therapeutic strategies in a disease-specific manner.

Although several researchers have observed promising evidence of disease-induced BBB permeability alterations using various imaging modalities,^11^^–^^15^ studies on disease-dependent heterogeneous BBB permeability across vascular types remain limited to date.^12^^,^^16^ Accurate quantification of BBB leakage has been hindered by several challenges, including (1) the need for high spatial resolution to resolve individual vessels, (2) the need for real-time assessment of extravasation dynamics, and (3) the ability to differentiate between arteries, capillaries, and veins. In general, *in vivo* noninvasive imaging modalities (e.g., MRI, PET, and SPECT)^11^^–^^15^ lack the spatial resolution necessary for single-vessel level analysis. *Ex vivo* confocal approaches (e.g., Evans blue, dextran, and NaFl)^17^^–^^20^ detect tracer accumulation after extravasation but are inherently limited in resolving the temporal dynamics of the leakage process.

*In vivo* two-photon live imaging is considered the only method that satisfies all the above-mentioned requirements. This technique has been validated in previous studies, demonstrating that BBB leakage can be directly observed at the individual vessel level, with clear evidence of arterial, capillary, and venous transcytosis of albumin and nanoparticles *in vivo*.^21^^,^^22^ This approach continues to be widely used for studying BBB function. It has also been applied to disease models such as Alzheimer’s disease, where it enables evaluation of microvascular dynamics and barrier properties under pathological conditions.^23^ Although *in vivo* two-photon imaging provides the necessary spatial and temporal resolution, interpreting resulting spatiotemporal datasets remains challenging due to their high dimensionality and data volume. Physical-model-based analyses can accurately describe diffusion on a microscale in homogeneous environments; however, modeling extravasation in brain tissue remains challenging due to its nonhomogeneous and complex structure, consisting of diverse cell types, heterogeneous extracellular and intracellular spaces, and intricate geometries.^24^ These features can lead to spatially varying diffusion coefficients along the blood–brain barrier interface and within the surrounding parenchyma.^25^ Moreover, given inherent limitations of fluorescence imaging (e.g., photobleaching, detector saturation, and scattering), fluorescence intensity does not always correlate linearly with tracer concentration.^26^^,^^27^ These nonlinear effects can hinder accurate interpretation in conventional fluorescence intensity-based analyses. These challenges necessitate alternative analytical strategies. To evaluate the extravasation pattern acquired through time series imaging, it is necessary to extract meaningful features that preserve the temporal structure of the data.^28^^–^^30^ Employing nonlinear dimensionality reduction methods such as uniform manifold approximation and projection (UMAP) can improve sensitivity in detecting subtle variations in complex imaging data. By capturing temporal and multidimensional relationships rather than relying solely on absolute intensity values, UMAP offers robustness against signal distortions and enables identification of vascular-level permeability patterns that might be overlooked by traditional intensity-based analyses.

To evaluate disease-specific vascular-type dependent BBB total leakage, we employed two well-established rodent models: pilocarpine-induced status epilepticus (SE) and lipopolysaccharide (LPS)-induced neuroinflammation (NI). These two disease models are known to show increased BBB total leakage.^20^^,^^31^^,^^32^ LPS is widely used to model neuroinflammation as peripheral LPS injection promotes systemic immune activation that subsequently triggers glial activation and endothelial dysfunction within the brain.^33^ In the SE model, seizure-induced neuronal hyperexcitability leads to BBB disruption primarily in arteriolar segments, driven by vascular smooth muscle hyperexcitability, impaired neurovascular coupling, and remodeling of cerebrovascular mural cells.^34^^–^^36^ By contrast, the NI model is driven by systemic immune activation that promotes leukocyte adhesion, cytokine-mediated endothelial activation, and junctional protein degradation, primarily affecting venules.^37^^–^^39^ Together, these models represent pathophysiologically distinct routes of BBB impairment, providing a valuable framework to compare how vascular-type-specific BBB total leakage varies across disease conditions. Given their distinct mechanisms of BBB disruption, we expected SE to primarily affect arterioles and NI to target venules. Accordingly, we developed a UMAP-based analysis pipeline for *in vivo* two-photon imaging to quantify single-vessel BBB total leakage across vascular types. This approach enabled sensitive and disease-specific detection of arterial and venous leakage patterns in SE and NI models, respectively.

## 2 Materials and Methods

### 2.1 Animals

All experimental procedures were conducted after obtaining approval from the Institutional Animal Care and Use Committee (IACUC) at Sungkyunkwan University. Male C57BL/6 mice (n=36, 8 to 12 weeks old; OrientBio, South Korea) were individually housed in a controlled environment maintained at 24°C to 25°C with a relative humidity of 50% to 60% and a 12-h inverted light/dark cycle (lights on at 9:00 pm). A subset of mice (n=11; Control, n=4; SE, n=4; NI, n=3) designated for *in vivo* two-photon imaging underwent cranial window installation surgery and were provided a 4- to 6-week recovery period in their home cages before imaging experiments. The remaining animals (n=13; Control, n=5; SE, n=4; NI, n=4) assigned to *ex vivo* imaging did not undergo surgical procedures and were maintained under identical housing conditions. After the recovery period, mice were randomly allocated to receive either pilocarpine to induce SE or LPS to model NI. An additional independent set of mice (n=12; Control, n=6; NI, n=6) was used solely for cytokine measurements (serum brain IL-1β and brain IL-1β) to validate the induction of neuroinflammation and was not included in the imaging experiments.

### 2.2 Status Epilepticus and Neuroinflammation Models

**Fig. 1:**
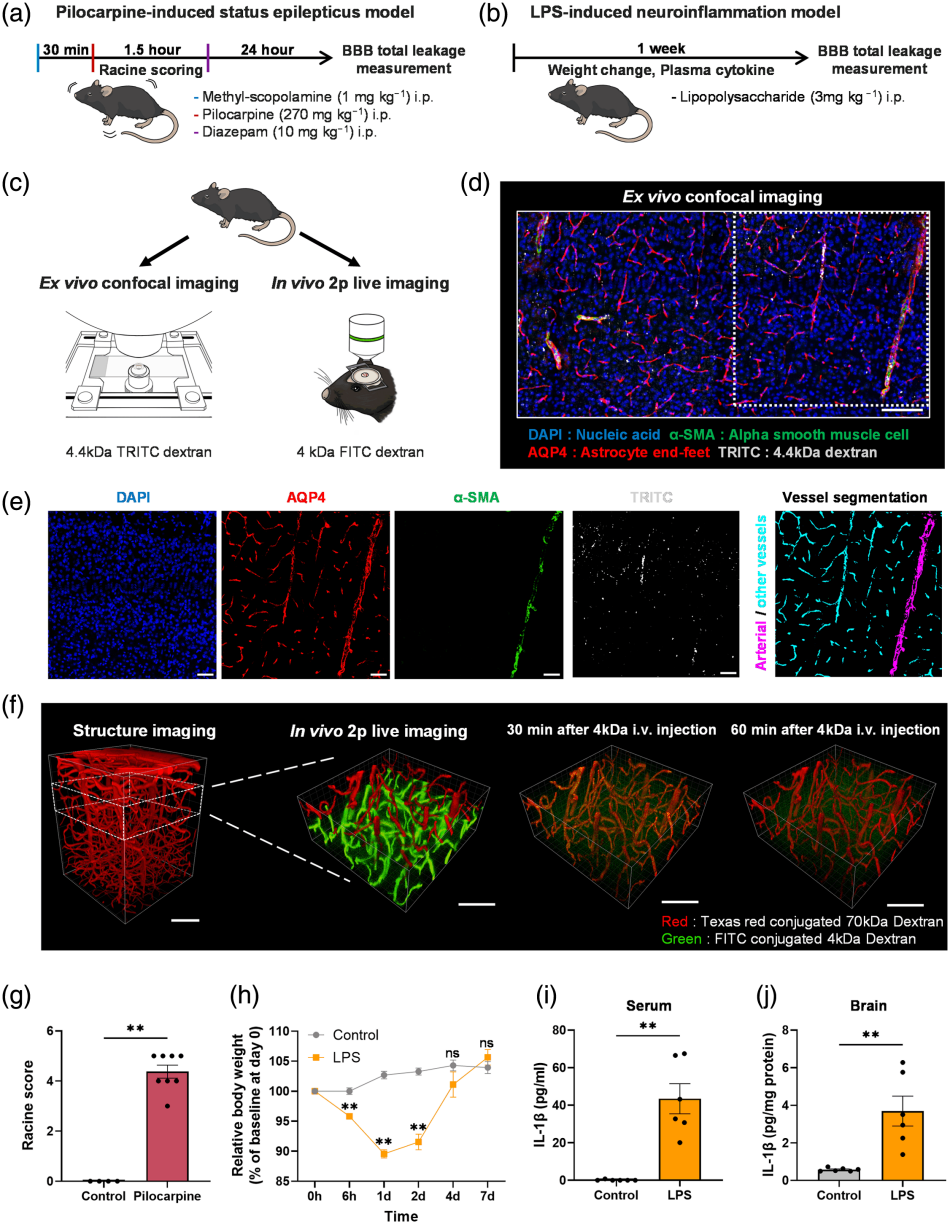
Experimental scheme and validation of disease models. (a) Schematic representation of the experimental timeline for the pilocarpine-induced status epilepticus model. (b) Schematic representation of the experimental timeline for the lipopolysaccharide (LPS)-induced neuroinflammation model. (c) Experimental setup for assessing blood–brain barrier (BBB) total leakage in both disease models. (d) Representative images of *ex vivo* confocal imaging. Scale bar: 100  μm. (e) Channel-separated views of the boxed region in (d) (DAPI, AQP4, α-SMA, TRITC) with overlays indicating vessel classification into α-SMA+ (arterial) vessels and α-SMA- (other types) vessels. Scale bar: 50  μm. (f) Representative images of *in vivo* two-photon (2p) live imaging. Scale bar: 100  μm. (g) Racine scores of control mice and pilocarpine-injected mice at 1.5 h post intraperitoneal (i.p.) injection (Control, n=4; Pilocarpine, n=8). (h) Relative body weight (% of baseline at day 0) following intraperitoneal injection of saline or LPS (Control: n = 5; LPS: n=7). Values are normalized to each animal’s initial weight (day 0 = 100%). (i) Comparison of serum IL-1β levels, a pro-inflammatory cytokine, between saline-injected and LPS-injected mice (Control, n=6; LPS, n=6). (j) Comparison of brain IL-1β levels, a pro-inflammatory cytokine, between saline-injected and LPS-injected mice (Control, n=6; LPS, n=6). Data are presented as mean ± SEM. Mann–Whitney U test. **, p<0.01; ns, not significant.

Mice were allowed to recover from cranial window surgery or adapt to their home cage environment for 4 to 6 weeks prior to induction of SE or NI. SE was induced by administering methyl-scopolamine (1  mg/kg, intraperitoneal [i.p.], Sigma-Aldrich, St. Louis, Missouri, United States) to minimize peripheral cholinergic effects, followed by administration of pilocarpine (270  mg/kg, i.p., Sigma-Aldrich) 30 min later. Continuous seizure activity was observed for 1.5 h, after which diazepam (10  mg/kg, i.p., SAMJIN, Anyang, South Korea) was administered to terminate seizures. Seizure severity was evaluated using a modified Racine scale ranging from stage 1 to stage 5 [Fig. 1(a)].^40^^–^^42^ Only animals that reached a Racine score of ≥3 were included in the SE group for subsequent analyses.

NI was induced by a single i.p. injection of LPS (3  mg/kg, strain O111:B4, Sigma-Aldrich), which promotes systemic immune activation and leads to neuroinflammatory responses in the brain. Body weight was monitored at 6 h, 1 day, 2 days, 4 days, and 7 days post-injection to assess physiological effects of LPS [Fig. 1(b)]. Only animals exhibiting a significant body-weight loss (≥5% from baseline within 48 hours) were included in the NI group for subsequent analyses. Subsequently, either *in vivo* two-photon imaging or *ex vivo* confocal imaging was performed to assess disease-related changes [Fig. 1(c)].

### 2.3 Cytokine Assessment

Blood samples were collected at 6 h after injection to measure systemic cytokine levels. Mice were anesthetized with Avertin (250  mg/kg). Whole blood was obtained via cardiac puncture into clot activator-coated tubes (Greiner Bio-One). Blood samples were allowed to clot at room temperature. They were then centrifuged at 5800 rpm for 15 min. Mouse ELISA kits (R&D Systems) were used to measure serum concentrations of TNF-α and IL-1β following the manufacturer’s protocol. Absorbance readings were obtained at 450 nm using a Synergy HT microplate reader (BioTek).

### 2.4 Measurement of BBB Total Leakage via *Ex Vivo* Confocal Imaging

For *ex vivo* confocal imaging, mice were anesthetized with isoflurane and intravenously injected via the tail vein with 4.4-kDa TRITC-conjugated dextran (10% dextran solution, 1.5  μL/g body weight, Sigma-Aldrich, St. Louis, Missouri, United States). At 1 h post-injection, mice were deeply anesthetized with Avertin (250  mg/kg, i.p.) and transcardially perfused with phosphate-buffered saline (PBS) using a peristaltic pump (P-1500, Harvard Apparatus, Holliston, Massachusetts, United States). Their brains were carefully extracted. Immunohistochemical staining was performed prior to *ex vivo* confocal imaging.

### 2.5 Immunohistochemical Staining

Following imaging, mice were deeply anesthetized with Avertin (250  mg/kg, i.p.) and transcardially perfused with PBS, followed by treatment with 4% paraformaldehyde (PFA). Extracted brains were post-fixed in 4% PFA for 24 h and then cryoprotected in 30% sucrose solution (dissolved in PBS) for 3 days. Tissues were embedded in a freezing medium (FSC22, Leica Biosystems, Wetzlar, Germany), frozen, and sectioned coronally at a thickness of 40  μm using a cryostat (CM1950, Leica Biosystems). Brain sections were incubated with the following primary antibodies: For *ex vivo* extravasation measurement, FITC-conjugated mouse anti-α-SMA (1:200, Sigma-Aldrich), and rabbit anti-AQP4 (1:200, Sigma-Aldrich). For tight-junction protein analysis: FITC-conjugated mouse anti-α-SMA (1:200, Sigma-Aldrich), goat anti-CD31 (1:500, R&D systems), and rabbit anti-ZO-1 (1:1000, Abcam). After washing with PBS, sections were incubated with the following secondary antibodies: Alexa Fluor 647-conjugated anti-rabbit IgG (1:200, A-31573, Invitrogen, Thermo Fisher Scientific) and Alexa Fluor 555-conjugated anti-goat IgG (1:350, A-21082, Invitrogen, Thermo Fisher Scientific). Following immunostaining, sections were mounted with DAPI-containing mounting medium (H-1500, Vector Laboratories, Newark, California, United States) and covered with a cover glass. Prepared slides were stored at 4°C until confocal imaging.

### 2.6 Confocal Microscopy

Fluorescent images were acquired using a confocal laser scanning microscope equipped with white light lasers (TCS SP8, Leica Microsystems CMS GmbH, Mannheim, Germany). Z-stack images were captured with a 40× water-immersion objective lens (numerical aperture 1.4). Excitation wavelengths of 405, 488, 555, and 647 nm with the same objective were used for imaging. For *ex vivo* extravasation measurement: DAPI, α-SMA+ smooth muscle cells, TRITC-conjugated 4.4-kDa dextran (injected intravenously via the tail vein), and AQP4+ vasculature, respectively. For tight junction protein analysis: DAPI, α-SMA+ smooth muscle cells, CD31+ vasculature, and ZO-1+ tight junction protein, respectively. All image acquisition fields were selected to include cortical layers 2 to 3, corresponding to regions analyzed by *in vivo* two-photon imaging. For both imaging sets (DAPI, α-SMA, TRITC, and AQP4; or DAPI, α-SMA, CD31, and ZO-1), regions containing α-SMA+ penetrating vessels were specifically targeted. Images were collected using a four-tile mosaic (2×2 configuration). Each tile was acquired at a resolution of 1024×1024  pixels with a spatial sampling of $0.18\times 0.18\times 1\;\mu m^{3}$ (x,y,z) [Figs. 1(d) and 1(e)].

### 2.7 Chronic Cranial Window Surgery for *In Vivo* Two-photon Live Imaging

To enable *in vivo* two-photon live imaging, mice underwent chronic cranial window surgery. Anesthesia was induced with 3% isoflurane and maintained at 1.5% throughout the procedure (MIP Company, Portland, Oregon, United States). Body temperature was kept at 37°C using a homeothermic heating pad system with rectal probe feedback (FHC, Bowdoin, Maine, United States). Heart rate and blood oxygen saturation (${\mathrm{SpO}}_{2}$) were continuously monitored using a physiological monitoring system (PhysioSuite, Kent Scientific, Torrington, Connecticut, United States) equipped with a mouse paw pulse oximeter sensor (Kent Scientific, Torrington, Connecticut, United States). Group-wise traces and means for heart rate and ${\mathrm{SpO}}_{2}$ are shown in the Supplementary Materials as Fig. S1 and confirm physiological stability across groups. Animals were secured in a stereotaxic frame (David Kopf Instruments, Los Angeles, California, United States) during surgery. A 3-mm-diameter circular craniotomy was performed over the right hemisphere, targeting a region devoid of large pial vessels. A custom-designed chamber frame (Narishige, Tokyo, Japan) was affixed to the skull using cyanoacrylate adhesive. The exposed cortical area was overlaid with a 4-mm glass coverslip (Warner Instruments, Hamden, Connecticut, United States) and sealed immediately using cyanoacrylate glue. The remaining space between the coverslip and skull was filled with dental resin to stabilize the window. Postoperatively, mice received intraperitoneal injections of enrofloxacin (Baytril, antibiotic) and meloxicam (Metacam, anti-inflammatory/analgesic). Animals were then allowed to recover in their home cages for 4 to 6 weeks to minimize potential neuroinflammatory effects prior to imaging sessions. Only animals that exhibited clear and stable recovery following the cranial window surgery were included for subsequent *in vivo* two-photon imaging.

### 2.8 Measurement of BBB Total Leakage via *In Vivo* Two-photon Live Imaging

Mice were anesthetized with 1.5% isoflurane and placed on a head-fixation apparatus (MAG-1, Narishige, Japan) positioned beneath a two-photon microscope. Body temperature of each mouse was maintained at 37°C using a homeothermic heating pad system with rectal probe feedback (FHC, Bowdoin, Maine, United States). To visualize vascular structure, Texas Red-conjugated 70-kDa dextran (2.5% solution; Sigma-Aldrich) was administered via tail vein injection at 1.5  μL/g body weight. Imaging was performed using a Ti:Sapphire femtosecond pulsed laser tuned to 950 nm (Chameleon Vision II, Coherent, Inc., Santa Clara, California, United States). Emitted fluorescence signals were collected using hybrid detectors (HyD) through a 525/50  nm bandpass filter for FITC-conjugated 4-kDa dextran and a 624/40  nm bandpass filter for Texas Red. Images were acquired with a 25×/0.95 NA water-immersion objective lens (HCX IRAPO), covering a field of view (FOV) of $354.29\times 354.29\;\mu m^{2}$ (512×512  pixels, 0.692  μm/pixel). Imaging was conducted at depths up to 400  μm from the cortical surface with a z-step size of 2  μm. Following structural imaging, a 3D imaging plane was re-identified at a depth of 50 to 100  μm to capture time-lapse images. FITC-conjugated 4-kDa dextran (5% solution; Sigma-Aldrich) was then injected intravenously at 1.5  μL/g body weight. Time-lapse z-stack images (512×512×40; $354.29\times 354.29\times 80\;\mu m^{3}$) were acquired every 15 s for 60 min [Fig. 1(f)].

### 2.9 Quantification Methods for *Ex Vivo* Confocal Imaging

Image preprocessing and quantitative analysis were performed using Fiji (ImageJ) and custom-written MATLAB scripts (MathWorks, Natick, Massachusetts, United States).

#### 2.9.1 Image preprocessing

Following confocal acquisition, images were first smoothed using a 3D Gaussian filter with a full width at half-maximum (FWHM) of 0.36  μm to reduce background noise and normalize vessel morphology. Background subtraction using a rolling ball algorithm was applied only to vessel structure images to enhance contrast for vessel segmentation and classification and was not applied to images used for quantitative leakage analysis to avoid potential distortion or loss of subtle fluorescence halos. For maximum intensity projection (MIP), all image stacks were cropped to a 15-μm thickness along the z-axis.

#### 2.9.2 Vascular type classification and dextran quantification

AQP4-positive vessels were binarized using a local thresholding method to quantify extravasation patterns of TRITC-conjugated 4.4-kDa dextran by vascular type. Vessels were classified as α-SMA+ (arterial vessels) or α-SMA- (other vessels) based on α-SMA signal. To define the perivascular region while preserving a consistent region of interest (ROI), the binarized AQP4+ vasculature was dilated by 15  μm using a disk-shaped kernel. The perivascular zone was calculated by subtracting the original vasculature mask from the dilated mask. The TRITC signal was binarized using local thresholding. Extravasation was quantified as the ratio of the area positive for TRITC signal (TRITC+ area) to the total area of the corresponding perivascular region for each vascular type. Specifically, within the defined perivascular ROI surrounding each vessel type.

#### 2.9.3 Quantification of ZO-1 immunoreactivity

ZO-1 immunoreactivity was quantified as the mean signal intensity (arbitrary units, A.U.) within CD31-defined vascular ROIs. To extract vessel boundaries, CD31-positive structures were binarized using a local thresholding method. Vessels were further classified into α-SMA+ (arterial vessels) or α-SMA- (other vessels) categories based on α-SMA signal. Quantification was performed separately for each vascular type.

### 2.10 Quantification Methods for *In Vivo* Two-Photon Imaging

Image preprocessing and quantitative analysis were conducted using IMARIS 8.2 (Bitplane, Schlieren, Switzerland), Fiji (ImageJ), and custom-written MATLAB scripts (MathWorks, Natick, Massachusetts, United States).

#### 2.10.1 Image preprocessing

To enhance the signal-to-noise ratio, both structural and time-lapse images were first smoothed using a 3D Gaussian filter with a FWHM of 1  μm. Background subtraction using a rolling ball algorithm was applied only to vessel structure images to improve contrast for vessel segmentation and classification and was not applied to images used for quantitative leakage analysis to avoid potential distortion or loss of subtle fluorescence halos. To correct for inhomogeneous intensity distribution in the x to y plane, preprocessed images were normalized by dividing them by a smoothed MIP image. This step was applied only to structure images to improve uniformity for vessel segmentation and was not applied to images used for quantitative leakage analysis so that true spatial variations in extravasation were preserved. This reference MIP was generated by excluding apical pial vessels and applying a 3D Gaussian filter with a 20-μm FWHM to estimate the intensity nonuniformity field [Figs. S2(a) and S2(b) in the Supplementary Material]. For longitudinal analysis, time-lapse frames were registered to structural images using an affine 3D transformation to ensure spatial alignment.

#### 2.10.2 Vascular type classification and perivascular region definition

Vascular structures were segmented using a trainable WEKA segmentation plugin in Fiji,^43^ which could integrate various machine learning algorithms. Morphological parameters, including vessel diameter and branching topology, were extracted from binarized images. Vessel diameters were calculated by generating a Euclidean distance map from the vessel centerline to its boundary. A 3D skeleton image was produced using Fiji’s skeletonize plugin and converted into a vascular network graph using a 26-neighbor voxel connectivity model [Figs. S2(c) and S2(d) in the Supplementary Material]. These diameter and skeleton maps were then merged to form a diameter-mapped vascular network. Arteries and veins were manually identified based on red blood cell (RBC) velocity and spontaneous diameter fluctuation observed during live imaging [Fig. S2(e) in the Supplementary Material]. Using this reference, the entire penetrating vascular tree was traced from the diameter-mapped graph and classified hierarchically into 0th-order (main penetrating vessels), first-order, and second-order branches. The 0th-order vessel was defined as the thickest segment oriented vertically to the imaging plane. Branching vessels were assigned based on their connections to the parent segment [Fig. S2(f) in the Supplementary Material]. All penetrating vessels up to the second order were further classified as arterial or venous based on the manually labeled parent vessel. Vessels with diameters <6  μm that were not classified as part of the arterioles or venules were considered capillaries. To define perivascular regions and minimize signal contamination, the binarized vasculature was spherically dilated by 5 and 15  μm. The perivascular space was defined as the voxel region between these two dilations. The inner boundary (5  μm) minimized fluorescence from intravascular signal and reduced potential effects of tissue geometry changes during time-lapse imaging, such as x to y drift of the vessel lumen into the measurement area, whereas the outer boundary (15  μm) reduced contamination from nearby vessels.^44^ This perivascular mask was applied to all segmented vessel types and uniquely paired with each vessel segment for subsequent analysis [Figs. S2(g) and S2(h) in the Supplementary Material].

#### 2.10.3 BBB leakage indices

To quantify vascular-type-specific BBB leakage across control, SE, and NI groups, previously defined perivascular regions were used as masks. Time-lapse fluorescence intensity changes were measured within each perivascular mask corresponding to individual vessel segments. Each segment was categorized as arterioles, capillaries, or venules based on prior vascular classification. For each vascular type, intensity changes were extracted as mean values over time from their respective perivascular regions. From these traces, several quantitative leakage metrics were computed, including area under the curve (AUC), average fluorescence intensity change relative to baseline ($\Delta F/F_{0}$; measured at 50 to 60 min), and averaged differential coefficient (ΔF/Δt). These indices represent conventional intensity-based leakage quantification approaches, which can summarize the magnitude of extravasation over time [Fig. S3(a) in the Supplementary Material]. In addition, UMAP distance was calculated to capture overall patterns in extravasation dynamics [Fig. S3(b) in the Supplementary Material]. To project individual vessel extravasation profiles into a two-dimensional UMAP space, UMAP version 2.2 (MATLAB implementation) was used with the following parameters: metric = Euclidean, n_neighbors = 5, and min_dist = 0.4. In UMAP, n_neighbors controls how much local versus global structure is preserved, with a lower value emphasizing a local relationship. min_dist determines how closely vessels with similar extravasation patterns are positioned in the low-dimensional space. These hyperparameters were selected because the resulting UMAP distance showed a strong positive correlation with conventional leakage indices, including AUC, $\Delta F/F_{0}$, and ΔF/Δt. UMAP distance was defined as the average Euclidean distance between UMAP coordinates of each individual vessel and those of vessels in the control group. The diagnostic sensitivity of each BBB leakage index was assessed by calculating the area under the receiver operating characteristic curve (ROC-AUC) value for each vascular type, enabling evaluation of both reliability and discriminative power of extracted metrics. For both the conventional and UMAP-based analyses, all measured vessel segments were included in the statistical analysis. In this study, the term leakage index collectively refers to these quantitative metrics (AUC, $\Delta F/F_{0}$, ΔF/Δt, and UMAP distance) that characterize BBB leakage dynamics in a vessel-type-specific manner.

### 2.11 Measurement of RBC Velocity via *In Vivo* Two-Photon Live Imaging

RBC velocity was measured *in vivo* using two-photon laser scanning microscopy. The vascular lumen was labeled by intravenous injection of high-molecular-weight fluorescein-conjugated dextran, thereby allowing nonfluorescent RBCs to appear as dark shadows against the fluorescent plasma background. Imaging was performed with a 25×/0.95  NA water-immersion objective lens (HCX IRAPO, Leica) at zoom 6. Line scan imaging was conducted out along the central axis of cortical vessels (pixel width: 0.144  μm) at a scan rate of 2000 Hz, corresponding to 0.5 ms per line.

Line scan images were converted into space–time plots, in which the movement of RBCs generated diagonal streaks, the angle of which reflected their velocity. To estimate the streak angle, Radon-transform-based image analysis was employed.^45^^–^^47^ Briefly, the images were divided into sliding time windows, and for each window, the local angle of RBC streaks was determined using the Radon transform after Sobel filtering to enhance streak edges. The velocity was then computed from the measured streak angle, spatial resolution, and scan rate according to established formulas.^45^^,^^47^ All image acquisition and analysis were performed using custom-written routines in MATLAB, following previously published procedures.^48^

### 2.12 BBB Permeability Analysis

To complement the analysis of total leakage, we further calculated the volume transfer constant ($K_{\mathrm{trans}}$) using Patlak linear regression between peri-vascular and intra-vascular fluorescence signals.^49^ To account for flow-related effects, we derived a flow-normalized $K_{\mathrm{trans}}$ by incorporating vessel cross-sectional area and RBC velocity (with hematocrit fixed at 0.45) into the Renkin–Crone model.^50^^,^^51^ This procedure allowed correction for physiological differences in blood flow and vascular geometry across vessel types (arterioles, capillaries, and venules) and conditions (Control, SE, NI).

### 2.13 Statistical Analysis

All statistical analyses were conducted using IBM SPSS Statistics (SPSS Inc., Chicago, Illinois, United States) or GraphPad Prism 9.5.1 (GraphPad Software, San Diego, California, United States). The normality of data distribution was assessed with the Shapiro–Wilk test. Nonparametric statistical methods were used when the distribution of data within a group deviated from normality. For comparisons between two groups with a single dependent variable, Student’s t-test or Mann–Whitney U test was conducted. In cases of three-group comparisons, one-way ANOVA or Kruskal–Wallis test was used to determine overall group differences, followed by Bonferroni-corrected post hoc analyses. Diagnostic potential of the index for disease was assessed using an ROC curve analysis.

## 3 Results

### 3.1 Validation of Disease Models

To validate the pilocarpine-induced SE model, behavioral seizures were scored using a modified Racine scale, with severity ranging from stage 1 to stage 5. Mice in the SE group exhibited significantly higher Racine scores compared to controls (p<0.0001), confirming successful induction of status epilepticus [Fig. 1(g)]. For the LPS-induced NI model, disease validation was performed by monitoring body weight and serum pro-inflammatory cytokine levels. LPS-injected mice showed a marked reduction in body weight over the first two days of post-injection relative to controls [Fig. 1(h), p=0.004]. In addition, serum concentrations of IL-1β were significantly elevated in the NI group compared to the control group at 6 hours post-injection [Fig. 1(i), p=0.0022], indicating an evident systemic inflammatory response. Consistent with this, brain IL-1β levels were also significantly increased in the NI group at 6 hours post-injection [Fig. 1(j), p=0.0022], further supporting the induction of neuroinflammation.

### 3.2 *Ex Vivo* Confocal Imaging for Assessing Heterogeneous BBB Total Leakage Change

**Fig. 2:**
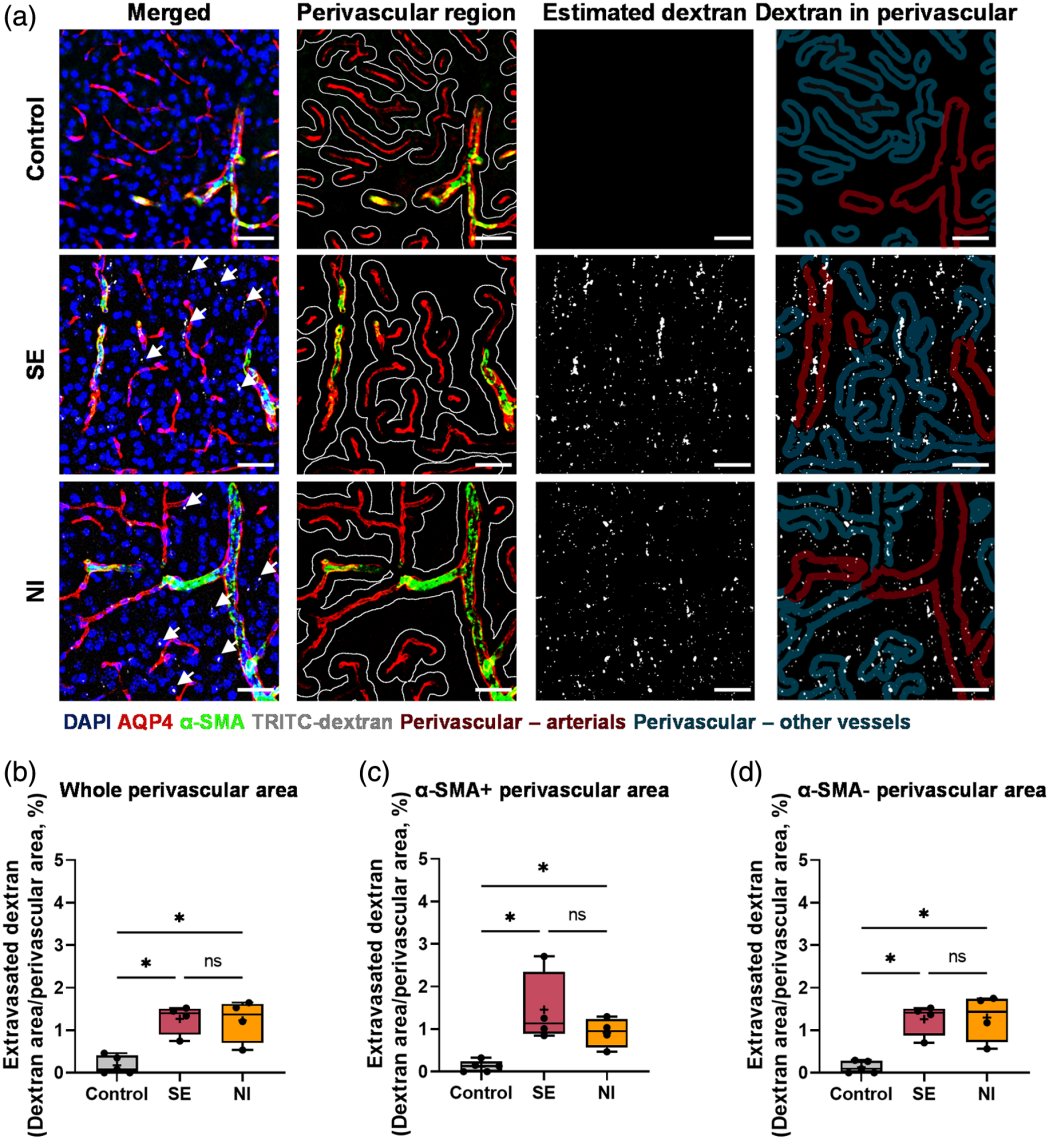
*Ex vivo* confocal imaging and quantification of TRITC-dextran leakage at 1 h after intravenous (i.v.) administration of 4.4 kDa TRITC-dextran. (a) Representative confocal images illustrate TRITC-dextran leakage in Control, SE, and NI groups. The perivascular area was defined as a 15  μm region surrounding AQP+ vessels (white line). α-SMA staining identified perivascular areas associated with different vessel types (red and blue shaded regions). AQP+ signal was used to remove the intravascular signal and exclude perfusion artifacts. Scale bar: 50  μm. (b–d) Quantification of TRITC-dextran leakage: (b) ratio of TRITC-dextran signal to total perivascular area. (c) Ratio of TRITC-dextran signal to α-SMA+ perivascular area. (d) Ratio of TRITC-dextran signal to α-SMA-perivascular area. Control, n=5; SE, n=4; NI, n=4. Median values are indicated by black lines, and mean values by “+” symbols. Kruskal–Wallis test with Bonferroni-corrected post hoc analyses was used for multiple comparisons. *, p<0.05; ns, not significant.

To assess heterogeneous BBB total leakage using *ex vivo* confocal imaging, 4.4-kDa TRITC-conjugated dextran was administered intravenously and analyzed in brain tissue sections at one hour post-injection. As shown in Fig. 2(a), representative images illustrated an increase in accumulation of TRITC in both disease groups compared to controls. Quantitative analysis confirmed this trend, showing significantly greater TRITC accumulation in the perivascular region across all vessels (mean ± SEM of % TRITC+ area per perivascular area: 0.18±0.10 for the control; 1.27±0.18 for SE, p=0.016; 1.23±0.25 for NI, p=0.016) [Fig. 2(b)], showing no significant difference between disease groups (p=0.886). To evaluate leakage changes based on vascular type, vessels were categorized as α-SMA+ or α-SMA−. TRITC accumulation was then analyzed accordingly [Figs. 2(c) and 2(d)]. Both disease groups exhibited increased TRITC accumulation in the perivascular regions of α-SMA+ vessels (mean ± SEM, %: 0.12±0.06 for control; 1.46±0.43 for SE, p=0.016; 0.92±0.17 for NI, p=0.016) and α-SMA− vessels (mean ± SEM, %: 0.13±0.06 for control; 1.26±0.19 for SE, p=0.016; 1.30±0.28 for NI, p=0.016) compared to controls. No significant difference was observed between the SE and NI groups for either α-SMA+ (p=0.686) or α-SMA−(p=0.886) perivascular regions.

### 3.3 *In Vivo* Two-photon Imaging for Assessing Heterogeneous BBB Total Leakage Change

**Fig. 3:**
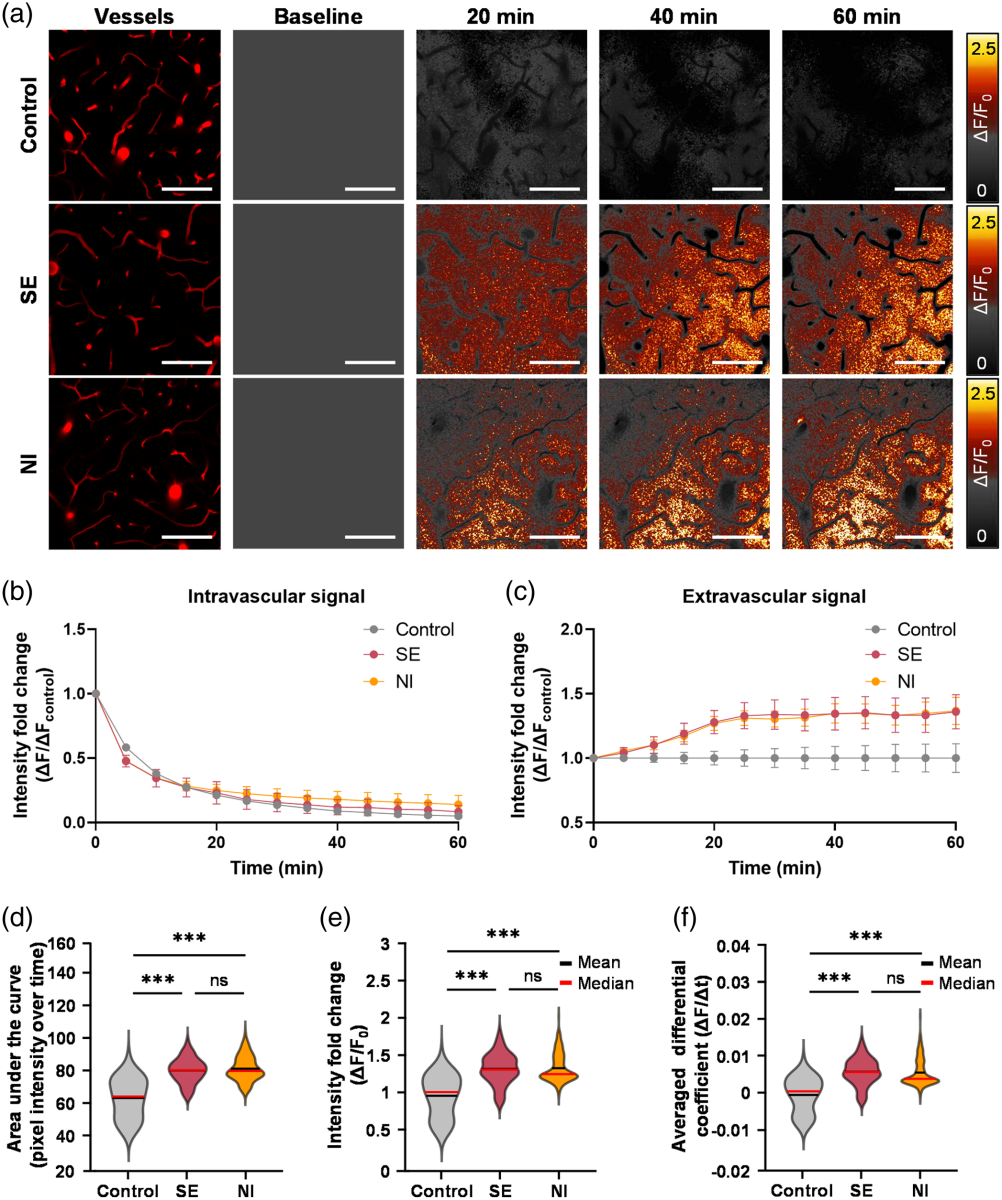
Increased blood–brain barrier (BBB) total leakage in the whole parenchymal region following disease induction. (a) Representative images of fluorescence-labeled dextran leakage. The left panel displays the original, unprocessed images, whereas the right panel visualizes the same data as a time-lapse sequence using a color-coded intensity scale. Scale bar: 100  μm. (b) and (c) show changes in fluorescence intensity in intravascular and extravascular regions, respectively. Extravascular fluorescence intensity was elevated in both SE and NI groups, whereas no significant difference was observed in the intravascular region between groups. (d–f) show quantitative metrics derived from the extravascular fluorescence intensity in (c): (d) Area under the curve (AUC), (e) Intensity fold change ($\Delta F/F_{0}$), and (f) averaged differential coefficient (ΔF/Δt). Control, n=4 (615 vessels); SE, n=4 (539 vessels); NI, n=3 (497 vessels). Mean values are indicated by black lines, and median values are indicated by red lines. Data are presented as mean ± SEM. One-way ANOVA followed by Bonferroni-corrected post hoc analyses was used for multiple comparisons. ***, p<0.001; ns, not significant.

To evaluate whether two-photon imaging could effectively detect increased BBB total leakage in NI and SE models, *in vivo* imaging was conducted following intravenous administration of 4-kDa FITC-dextran via a tail vein catheter. Live imaging immediately after injection revealed increased extravasation of FITC-dextran in both disease groups over time, whereas no extravasation was observed in the control group for 60 min [Fig. 3(a)]. Despite comparable intravascular fluorescence intensities between groups [Fig. 3(b)], extravasated FITC-dextran intensity at 60 min was 1.4-fold higher in the disease groups than in controls [Fig. 3(c)]. This indicated that although 4-kDa dextran exhibited similar intravascular kinetics, it extravasated into the parenchyma due to BBB disruption in disease conditions. To quantify and compare extravasation levels, leakage indices including AUC, $\Delta F/F_{0}$, and ΔF/Δt were calculated. As shown in Figs. 3(d)–3(f), all indices demonstrated a significant increase in BBB total leakage in the disease groups compared to controls (Tables S1 and S2 in the Supplementary Material, p<0.001). However, no significant differences were observed between NI and SE models (AUC: p=0.669; $\Delta F/F_{0}$: p=0.214; ΔF/Δt: p=0.082). These results suggest that although whole-parenchyma leakage indices could effectively detect overall BBB dysfunction, they could not capture disease-specific leakage differences.

**Fig. 4:**
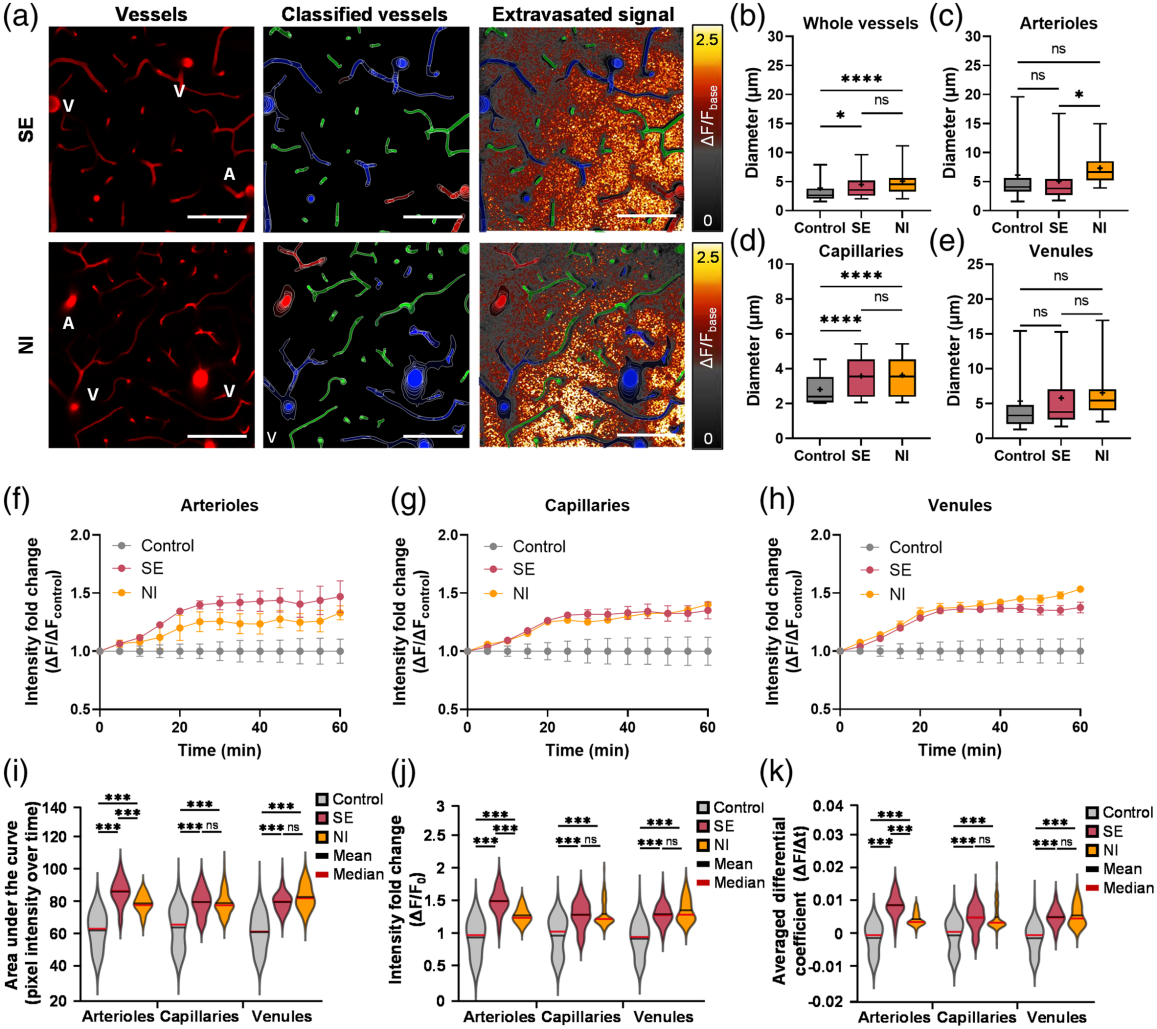
Heterogeneous BBB total leakage in SE and NI models. (a) Representative images of fluorescence-labeled dextran extravasation, showing vascular-type dependence. The left panel shows the vascular structure. Vasculature is color-coded: arterioles (red), capillaries (green), and venules (blue). Time-lapse data represented using a color intensity scale are shown in the right panel. The extravasated signal revealed vascular-specific patterns. The SE group exhibited enhanced extravasation in arterioles, whereas the NI group showed enhanced extravasation in venules. Scale bar: 100  μm. (b–e) Vessel diameter information in Control, SE, NI. (b) Whole vessels. (c) Arterioles. (d) Capillaries. (e) Venules. Quantification of extravasation in (f) arterioles, (g) capillaries, and (h) venules. In agreement with representative images, quantitative analysis of extravasation revealed increased arterial leakage in the SE group and enhanced venous leakage in the NI group. (i) Area under the curve (AUC), (j) Intensity fold change ($\Delta F/F_{0}$), and (k) averaged differential coefficient (ΔF/Δt). Control, n=4 (39 arterioles, 383 capillaries, 193 venules); SE, n=4 (47 arterioles, 323 capillaries, 169 venules); NI, n=3 (44 arterioles, 285 capillaries, 168 venules). Box plot: Median values are indicated by black lines, and mean values by “+” symbols. Violin plot: Mean values are indicated by black lines, and median values are indicated by red lines. Data are presented as mean ± SEM. One-way ANOVA followed by Bonferroni-corrected post hoc analyses was used for multiple comparisons. *, p<0.05; ***, p<0.001; ****, p<0.0001; ns, not significant.

To further investigate single-vessel leakage properties, extravasation patterns were analyzed across vascular subtypes (e.g., arterioles, capillaries, and venules). Time-lapse images merged with vessel classification [Fig. 4(a)] revealed distinct extravasation patterns for each disease model, with SE showing arteriolar-enriched leakage and NI showing venule-enriched leakage. Based on these observations, we systematically assessed vascular-type-dependent BBB total leakage by averaging single-vessel extravasation dynamics within defined perivascular regions [Figs. S2(g) and S2(h) in the Supplementary Material) and compared them across vessel classifications. Because vessel geometry can modulate leakage readouts, we next compared vessel diameters across groups [Figs. 4(b)–4(e)]. The whole-vessel diameter distribution was significantly increased in SE and NI compared to Control (mean ± *SD*, μm: 3.87±5.20 for Control; 4.52±3.58 for SE, p=0.0417; 5.09±3.15 for NI, p<0.0001) [Fig. 4(b)]. Arteriolar diameter was significantly greater in NI than in SE (p=0.0229) (mean ± *SD*, μm, arterioles: 6.13±5.09 for Control; 5.08±4.04 for SE; 7.33±3.50 for NI) [Fig. 4(c)]. Capillary diameter was significantly greater in SE and NI than in Control (mean ± *SD*, μm, capillaries: 2.90±0.95 for Control; 3.60±1.12 for SE, p<0.0001; 3.69±1.19 for NI, p<0.0001) [Fig. 4(d)]. Venular diameter showed no significant differences across the three conditions (mean ± *SD*, μm, veins: 5.37±8.56 for Control; 5.81±5.19 for SE; 6.56±3.95 for NI; p=0.2458) [Fig. 4(e)].

As shown in Figs. 4(f)–4(h), both disease groups exhibited increased 4-kDa dextran extravasation over 60 min post-injection compared to the control group, regardless of vascular type (mean ± SEM in fold change, arterioles: 1.47±0.13 for SE; 1.33±0.06 for NI; capillaries: 1.35±0.07 for SE; 1.40±0.02 for NI; venules: 1.38±0.05 for SE; 1.53±0.01 for NI). Representative images consistently showed distinct extravasation patterns between the two disease models. Specifically, extravasation was more pronounced in the arterial perivascular region of the SE group than in that of the NI group. Conversely, in the venous perivascular region, the NI group exhibited a higher degree of extravasation than the SE group.

To further quantify single-vessel extravasation patterns, AUC, $\Delta F/F_{0}$, and ΔF/Δt were computed as conventional intensity-based leakage indices. As shown in Figs. 4(i)–4(k), all indices demonstrated a significant increase in BBB total leakage across all vascular types in disease models compared to controls (Tables S1 and S2 in the Supplementary Material, p<0.001). Notably, the SE group exhibited a significantly higher BBB total leakage in arterioles than the NI group, which was well captured by conventional quantitative indices (p<0.001). Although the NI group showed a more pronounced increase in venules, this difference was not fully reflected by conventional quantitative indices (AUC: p=0.054; $\Delta F/F_{0}$: p=0.654; ΔF/Δt: p=0.642). To better delineate the disease-type- and vascular-type-dependent heterogeneity in BBB total leakage, we employed UMAP-based dimensionality reduction.

### 3.4 UMAP-Based Clustering Reveals Vascular-Type-Dependent BBB Total Leakage Changes

**Fig. 5:**
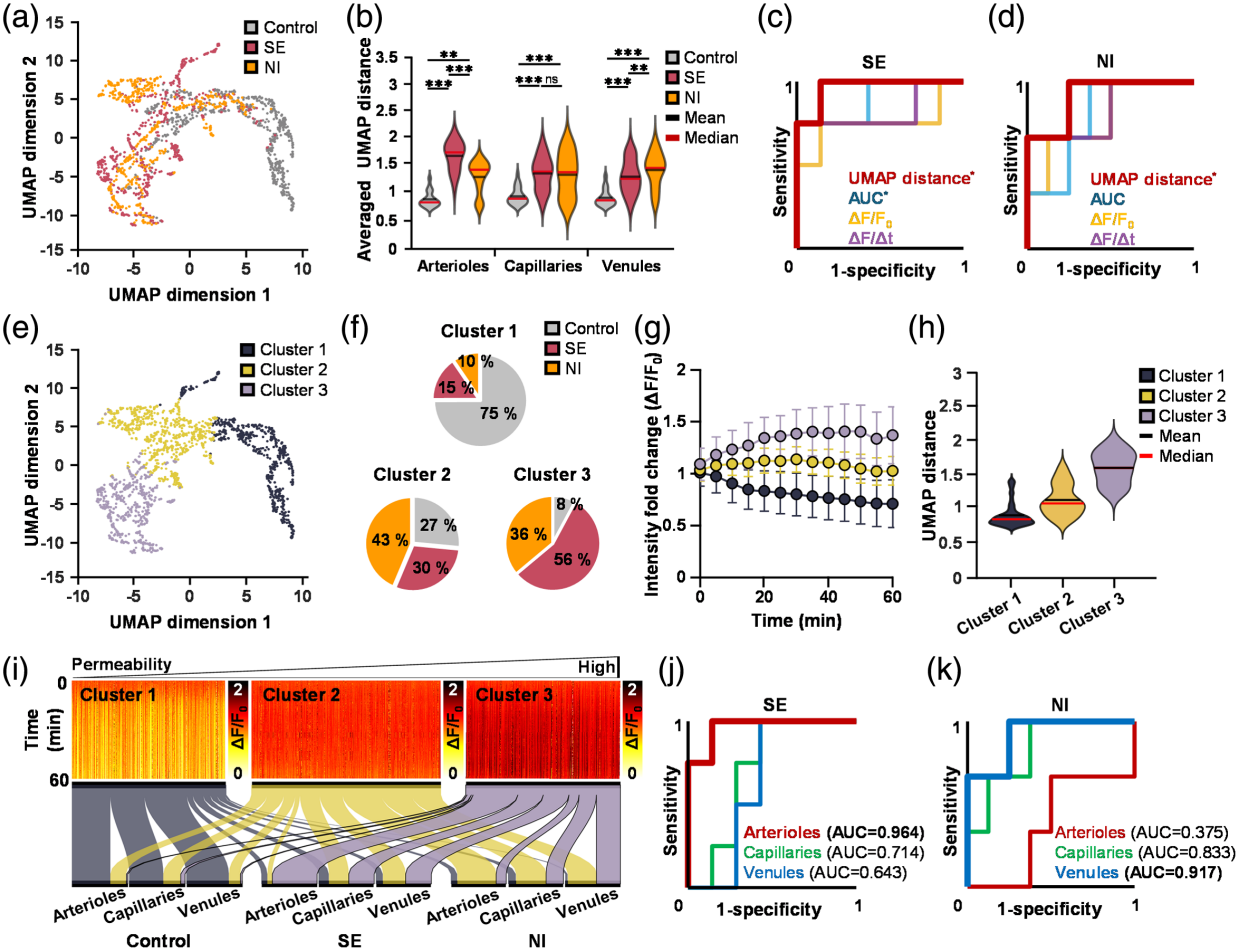
Uniform manifold approximation and projection (UMAP) and k-means clustering reveal vascular-type-dependent BBB total leakage patterns. (a) Representative scatter plot of normalized single-vessel extravasation pattern. Color indicates Control, SE, and NI groups. (b) Averaged UMAP distance in Control, SE, and NI group. Receiver operating characteristic (ROC) curve for diagnosing (c) SE group and (d) NI group. The ROC curve analysis revealed that UMAP distance exhibited the highest sensitivity among the evaluated BBB total leakage indices for distinguishing between the two disease models. (e) Representative scatter plot of normalized single-vessel extravasation pattern. Clusters 1, 2, and 3 were identified using k-means clustering. (f) Pie charts showing the proportion of each experimental group within each cluster, normalized to the number of single-vessel extravasation patterns per cluster. (g) Average single-vessel extravasation pattern for each cluster. (h) Average UMAP distance for each cluster. (i) Heatmap of single-vessel extravasation patterns clustered into three groups using k-means. Color intensity indicates extravasation intensity (see scale in right panel). Alluvial plot showing the proportion of vascular types within each cluster, normalized to the number of single-vessel extravasation patterns for each vascular type and group. ROC curves illustrate diagnostic performances for (j) SE and (k) NI groups. Consistent with proportions, the ROC curve showed that arterioles exhibited the highest sensitivity for diagnosing SE, whereas venules exhibited the highest sensitivity for diagnosing NI. Control, n=4 (39 arterioles, 383 capillaries, 193 venules); SE, n=4 (47 arterioles, 323 capillaries, 169 venules); NI, n=3 (44 arterioles, 285 capillaries, 168 venules). Mean values are indicated by black lines, and median values are indicated by red lines. Data are presented as mean ± SEM. One-way ANOVA followed by Bonferroni-corrected post hoc analyses was used for multiple comparisons. **, p<0.01; ***, p<0.001; ns, not significant.

To further explore single-vessel extravasation patterns, we projected individual vessel extravasation profiles into a two-dimensional UMAP space. A representative scatter plot of normalized single-vessel extravasation patterns [Fig. 5(a)] illustrated distinct clustering among Control, SE, and NI groups. Notably, UMAP distance analysis revealed a significant separation between SE and NI groups for venules (p=0.003), highlighting a vascular-type-dependent heterogeneity in BBB total leakage that was not fully captured by traditional leakage metrics [Fig. 5(b); Tables S1 and S2 in the Supplementary Material]. In addition, UMAP distance preserved the overall structure of single-vessel extravasation dynamics and demonstrated higher sensitivity in detecting subtle total leakage differences in venules than conventional leakage metrics.

To validate the sensitivity of variables, diagnostic performances of BBB total leakage indices were tested across the three groups using a ROC curve. Indices obtained from arterial data were used for SE diagnosis, whereas those from venous data were used for the NI group. As shown in Figs. 5(c) and 5(d), sensitivities of indices for diagnosing the SE group showed the following order (from high to low): UMAP (ROC-AUC = 0.964; p=0.014), AUC (ROC-AUC = 0.893; p=0.038), ΔF/Δt (ROC-AUC = 0.821; p=0.089), and $\Delta F/F_{0}$ (ROC-AUC = 0.750; p=0.186). For diagnosing the NI group, the sensitivity ranking showed the following result: UMAP (ROC-AUC = 0.917; p=0.041), ΔF/Δt (ROC-AUC = 0.833; p=0.102), $\Delta F/F_{0}$ (ROC-AUC = 0.792; p=0.153), and AUC (ROC-AUC = 0.792; p=0.153). Although conventional intensity-based indices (AUC, ΔF/Δt, and $\Delta F/F_{0}$) effectively distinguished arteria-specific total leakage changes in the SE group, the UMAP-based index further enhanced diagnostic sensitivity by providing more precise and consistent differentiation. In addition, UMAP effectively detected subtle vein-dominant differences in the NI model, which conventional indices failed to capture. Thus, morphometry-based analysis combined with UMAP dimensionality reduction offers a more robust, sensitive, and comprehensive approach, enabling reliable detection of both prominent and subtle vascular-type-dependent BBB total leakage alterations.

To further determine whether UMAP distance could reliably reflect extravasation pattern changes, we applied k-means clustering, an unsupervised learning algorithm, to objectively group similar single-vessel extravasation patterns based on their proximity in the UMAP embedding space [Fig. 5(e)]. This clustering approach provides a quantitative assessment of how effectively UMAP can capture extravasation dynamics. Three major clusters were identified. Cluster 1 contained predominantly control vessels (75%), whereas disease cases (SE and NI) became increasingly dominant in Cluster 2 (73%) and Cluster 3 (92%), indicating a progressive shift in vascular total leakage profiles across clusters [Fig. 5(f)]. Consistent with these distributions, extravasation levels and UMAP distances were the highest in cluster 3 (mean ± *SD* in fold change: 1.37±0.27 for $\Delta F/F_{0}$; 1.79±0.17 for UMAP distance), intermediate in cluster 2 (mean ± *SD* in fold change: 1.03±0.14 for ΔF/F0; 1.26±0.26 for UMAP distance), and the lowest in cluster 1 (mean ± *SD* in fold change: 0.71±0.14 for $\Delta F/F_{0}$; 0.99±0.23 for UMAP distance) [Figs. 5(g) and 5(h)]. These results support that UMAP embedding preserves biologically meaningful extravasation patterns. In addition, a 2D representation of single-vessel extravasation patterns [Fig. 5(i)] demonstrated distinct extravasation intensities across clusters. These findings confirmed that UMAP coordinates could preserve extravasation patterns and that the distances between points could reliably reflect extravasation differences.

Finally, we analyzed vascular-type distribution within each cluster. In the control group, arterioles (69%), capillaries (62%), and venules (77%) vessels were predominantly found in cluster 1. By contrast, their proportions were significantly lower in SE (arterioles: 11%, capillaries: 18%, venules: 13%) and NI (arterioles: 11%, capillaries: 14%, venules: 4%) groups. Proportions of vessels in clusters 2 and 3 (associated with increased extravasation) were significantly higher in both SE (arterioles: 89%, capillaries: 81%, venules: 88%) and NI (arterioles: 88%, capillaries: 85%, venules: 95%) groups than in the control group (arterioles: 31%, capillaries: 38%, venules: 23%). Notably, within cluster 3, arterioles in the SE group showed the highest proportion (81%), whereas venules in the NI group were the most prevalent (43%), further highlighting disease-specific vascular total leakage patterns [Fig. 5(i)]. These results suggest that pathological conditions can induce a vascular-type-specific shift toward more severe extravasation profiles, with arterioles primarily affected in SE, whereas venules were mainly affected in NI.

Consistently, the BBB total leakage index from arterioles showed the highest ROC-AUC among vascular-type-dependent indices for diagnosing SE [Fig. 5(j)]. Similarly, for diagnosing the NI group, the BBB total leakage index from venules exhibited the highest ROC-AUC among vascular-type-dependent indices [Fig. 5(k)]. These results indicate that BBB total leakage changes in a disease-specific manner depending on vascular type and/or density distribution.

## 4 Discussion

Although structural and molecular heterogeneity of the BBB has been extensively studied, direct assessments of heterogeneous BBB leakage within intact brain tissues remain limited. Accurate measurement of BBB leakage changes is critical for understanding disease progression and evaluating therapeutic efficacy. In this study, we directly compared *ex vivo* confocal imaging and *in vivo* two-photon imaging methods. Although *ex vivo* imaging identified overall increases of 4-kDa dextran extravasation in both disease models, it failed to distinguish disease-specific and vessel-type-specific total leakage differences. Conversely, *in vivo* two-photon imaging successfully captured distinct patterns of BBB total leakage, particularly highlighting significantly greater arterial extravasation observed in the status epilepticus (SE) model compared to the NI model. Quantitative analysis of total leakage using both traditional intensity-based indices (AUC, ΔF/Δt, $\Delta F/F_{0}$) and the UMAP-based method further confirmed that UMAP distance provided superior sensitivity compared to traditional indices in discriminating subtle, disease-dependent vascular total leakage variations. Thus, our proposed combination of *in vivo* two-photon imaging with UMAP dimensionality reduction offers a robust and sensitive analytical framework for assessing BBB total leakage heterogeneity at single-vessel resolution.

### 4.1 Advantages of *In Vivo* Two-photon Imaging Compared to *Ex Vivo* Confocal Imaging

To compare heterogeneous BBB total leakage assessment, we performed and compared *ex vivo* confocal and *in vivo* two-photon live imaging, carefully matching dextran molecular weight, disease induction methods, and experimental time points. Although both imaging methods detected increased BBB total leakage to 4-kDa dextran in SE and LPS models, *ex vivo* confocal imaging did not differentiate between the two disease models in terms of vascular-type-specific total leakage changes. By contrast, *in vivo* two-photon imaging revealed significantly higher extravasation in arterioles of the SE group than in the NI group, with a slight increase in venous total leakage observed in the NI group relative to the SE group. A key distinction between the two methods is the capacity to capture spatio-temporal dynamics. *Ex vivo* confocal imaging, by its nature, cannot observe real-time BBB leakage patterns or visualize the entire vasculature, including capillaries, arterioles, and venules. This technique is limited to detecting leaked tracer at a single time point after BBB passage. Consistent with this limitation, our *ex vivo* confocal data showed that TRITC-dextran was confined to less than 3% of the imaged area (Fig. 2), whereas FITC-dextran observed with *in vivo* two-photon imaging diffused throughout the parenchyma (Fig. 3). Furthermore, *ex vivo* confocal results can be influenced by protocol variations and timing,^17^^–^^20^^,^^34^ whereas *in vivo* two-photon imaging allows direct observation of blood vessel total leakage in living animals, providing accurate assessment of vascular abnormalities.^16^^,^^21^^,^^22^ In addition, *in vivo* two-photon imaging enables real-time observation of extravasation dynamics, reducing temporal bias by capturing continuous, intrinsic changes during the observation period. Consistently, using these properties, previous studies^21^^,^^22^ have reported experimental evidence showing that characteristics of transcytosis according to tracer could be observed in real-time for each vessel. In this study, we also propose a method for successfully capturing disease- and vessel-type-dependent changes in BBB total leakage by analyzing single-vessel extravasation patterns using *in vivo* two-photon live imaging.

### 4.2 BBB Total Leakage Indices

In this study, vascular-type-dependent BBB total leakage differences in both diseases were revealed by analyzing single-vessel extravasation patterns and calculating the distance in 2D embedded UMAP coordinates. UMAP, a nonlinear dimensionality reduction algorithm, can learn data structure and provide structure-preserved low-dimensional embeddings.^29^ To validate the reliability and sensitivity of UMAP distance, representative values of AUC, $\Delta F/F_{0}$, ΔF/Δt, and UMAP distance of single-vessel extravasation patterns were calculated and compared as BBB total leakage indices. A UMAP hyperparameter optimization using min_dist and n_neighbors was performed because hyperparameters could affect the embedding coordinate in UMAP dimension.^29^ Various UMAP distances showed strong positive correlations with AUC, $\Delta F/F_{0}$, and ΔF/Δt. Based on the correlation coefficient, the optimized hyperparameter was determined as min_dist = 0.4 and n_neighbors = 5. The UMAP distance calculated using the optimized hyperparameter showed the same tendency as all averaged representative values for each disease/vascular type. Single-vessel extravasation patterns included in each UMAP cluster also showed distinct patterns depending on the cluster, as evidenced by their UMAP coordinates [Figs. 5(e) and 5(g)]. These results indicate that UMAP distance can preserve single-vessel extravasation patterns. Thus, the UMAP distance could reflect single-vessel extravasation patterns without bias. This conclusion is consistent with recent studies showing that UMAP can preserve the real value as both local and global embeddings better than other dimensional reduction methods.^30^^,^^52^^,^^53^

The UMAP distance is not an absolute metric. Rather, it reflects the relative positioning of experimental subjects within a reduced-dimensional space.^29^^,^^54^ For this reason, it can capture subtle differences in high-dimensional extravasation patterns with greater sensitivity than traditional indices.^53^ Due to its higher sensitivity and reliability than other BBB total leakage indices, the proposed method could detect the difference of total leakage between SE and NI groups according to vascular type. Comparing disease-diagnostic ability using extracted BBB total leakage indices, UMAP has the highest disease-diagnostic ability among all indices. In addition, only UMAP was able to show the significant difference of the minutely different extravasation pattern in venules between groups. It means that UMAP shows high sensitivity in time series data analysis.^28^ Thus, UMAP distance not only exhibits a consistent tendency with traditional analysis methods but also provides an unbiased representation of single-vessel extravasation patterns. Furthermore, it can reliably capture disease- and vessel-type-dependent differences, outperforming conventional leakage indices in both sensitivity and discriminative power. However, because UMAP integrates subtle differences in local tracer dynamics, its output may not exclusively reflect endothelial properties. Instead, it may also be influenced by the surrounding microenvironment of each vascular niche. Such factors include the presence, distribution, and type of smooth muscle cells, the degree of pericyte coverage, astrocytic endfeet, perivascular macrophages, and the availability of perivascular space. Thus, although UMAP provides enhanced sensitivity and discrimination, its readout should be interpreted as a composite signature shaped by both endothelial leakage and these perivascular influences.

### 4.3 BBB Permeability

In this study, vascular-type- and disease-dependent BBB dysfunction was initially assessed by quantifying BBB total leakage, a metric that does not account for differences in blood plasma volume among vessel types and disease conditions. To more directly evaluate the intrinsic permeability of individual vessels under different pathological states, we further incorporated blood flow velocity, vessel diameter, and plasma volume into our calculations. We calculated permeability metrics ($K_{\mathrm{trans}}$^49^ and flow-normalized $K_{\mathrm{trans}}$^50^^,^^51^) by incorporating vessel geometry and RBC flow parameters. To incorporate flow parameters into our calculations, we performed additional experiments to measure RBC velocity for each vascular type across the three conditions (Control, SE, and NI). No significant differences were observed among groups for arterioles, capillaries, or venules (mean ± *SD*, mm/s: arterioles, 5.02±1.58 for Control, 5.57±2.53 for SE, 6.52±1.59 for NI; p=0.3076; capillaries, 1.05±0.52 for Control, 0.74±0.58 for SE, 0.82±0.37 for NI; p=0.0722; venules, 1.77±0.34 for Control, 1.38±0.56 for SE, 1.84±0.91 for NI; p=0.1769) [Fig. S4 in the Supplementary Materials].

When permeability was calculated, both SE and NI groups exhibited increased permeability across all vascular types compared to controls (SE, p<0.0001; NI, p<0.0001) [Figs. S5(a) and S5(c) in the Supplementary Materials], consistent with the previous total leakage quantification [Figs. 4(i)–4(k)]. Notably, the arterial-dominant pattern in SE remained robust when assessed by both total leakage and permeability metrics. Within SE, arterioles displayed significantly higher permeability than capillaries or venules ($K_{\mathrm{trans}}$: arterioles vs. capillaries, p=0.0116; arterioles vs. venules, p=0.0372; flow-normalized $K_{\mathrm{trans}}$: arterioles vs. capillaries, p<0.0001; arterioles vs. venules, p<0.0001) [Figs. S5(b) and S5(d) in the Supplementary Materials].

However, the vein-dominant increase in total leakage previously noted in the NI group was not preserved in permeability quantification [Fig. S5 in the Supplementary Materials]. With flow-normalized $K_{\mathrm{trans}}$, venular permeability was higher than that of capillaries (p=0.0005), but the difference from arterioles was not significant (p=0.8834) [Fig. S5(d) in the Supplementary Materials].

This discrepancy arises because total leakage and permeability capture distinct physiological processes. Total leakage accumulates the amount of tracer in the perivascular space over time and is subject to various influences such as local hemodynamics, vascular architecture, and clearance rates, which can diverge under pathological conditions. By contrast, $K_{\mathrm{trans}}$ and flow-normalized $K_{\mathrm{trans}}$ account for these variables by specifically quantifying the rate of transvascular tracer transfer, thus isolating intrinsic barrier permeability.

Therefore, although both metrics robustly identified the arterial-dominant BBB dysfunction in SE, the apparent vein-dominant leakage in NI observed with total leakage was not as evident in the permeability analysis, which may reflect the effects of correcting for underlying vascular and flow-related variables.

In summary, although some differences in trends were observed, our real-time single-vessel analysis revealed vascular-type- and disease-dependent BBB dysfunction through both BBB total leakage and BBB permeability measurements.

### 4.4 Vascular-Type-Dependent Differences in BBB Dysfunction in SE and NI Models

Our single-vessel analysis revealed that BBB total leakage occurs in a vascular-type-dependent manner, depending on the disease conditions. In the SE model, BBB total leakage increased predominantly in arterial segments, whereas the NI model exhibited leakage primarily in venules. Although the precise molecular mediators were not directly assessed in this study, the observed total leakage patterns are consistent with known vascular vulnerabilities in each condition.

In SE, seizure-induced neuronal hyperexcitability imposes substantial mechanical and metabolic stress on the cerebrovasculature. Arterioles are particularly susceptible due to their critical role in regulating cerebral blood flow. Intense seizure activity has been shown to disrupt arteriolar function through multiple converging mechanisms. First, mural cells, including pericytes and vascular smooth muscle cells (VSMCs), undergo structural remodeling that compromises vessel integrity.^34^ Second, seizures increase VSMC excitability, partly via TRPC3 channel activation, leading to pathological vasoconstriction that impairs neurovascular coupling and perfusion homeostasis.^35^ Finally, ultrastructural changes in arteriolar endothelium, including tight junction disassembly and increased transcytosis, contribute to barrier breakdown.^36^ Collectively, these findings suggest that arterioles serve as a primary locus of BBB vulnerability during seizure-driven neurovascular dysfunction.

By contrast, the NI model is characterized by systemic immune activation, resulting in elevated circulating cytokines that act on brain endothelial cells.^55^^,^^56^ These cytokines promote upregulation of adhesion molecules, junctional disruption, and endothelial activation, changes that are particularly pronounced at post-capillary venules. Due to their lower shear stress, looser junctional architecture, and higher baseline total leakage, venules are structurally predisposed to immune cell trafficking and inflammation-induced leakage.^39^^,^^57^ Consistently, leukocyte adhesion and trans-endothelial migration have been shown to occur predominantly at venular segments during systemic inflammation.^37^^,^^38^

To further support these findings, we performed immunohistochemical analysis of the tight junction protein ZO-1 [Fig. S6(a) in the Supplementary Materials]. Our results showed that ZO-1 expression was significantly reduced in both disease models relative to the control group (mean ± *SD*, A.U., 1±0.17 for Control; 0.69±0.10 for SE, p=0.048; 0.57±0.14 for NI, p=0.024) [Fig. S6(b) in the Supplementary Materials]. These results are consistent with the observed increases in both BBB total leakage and permeability in SE and NI compared to controls.

Stratifying the analysis by vascular subset, we found that ZO-1 expression in α-SMA+ (arterial) vessels was significantly decreased in both the SE and NI groups (mean ± *SD*, A.U., 1±0.19 for Control; 0.59±0.11 for SE, p=0.024; 0.54±0.17 for NI, p=0.048) [Fig. S6(c) in the Supplementary Materials]. This finding partially aligns with our total leakage and permeability measurement, which identified arterial-dominant leakage in SE. By contrast, ZO-1 expression in α-SMA- (capillary/venous) vessels was significantly decreased only in the NI group (mean ± *SD*, A.U., 1±0.20 for Control; 1.04±0.10 for SE; 0.66±0.04 for NI, p=0.024) [Fig. S6(d) in the Supplementary Materials]. This finding aligns with the venule-enriched total leakage observed in NI.

Together, these results indicate that BBB breakdown is not spatially uniform but instead shaped by disease-specific mechanisms that selectively compromise distinct vascular compartments. Our single-vessel imaging approach provides a sensitive platform for identifying such compartmentalized barrier disruptions, offering insights into vascular-type-specific pathophysiology in neurological disorders.

### 4.5 Application and Future Directions of the Proposed Method

Increased BBB total leakage is a pathological hallmark resulting from disease-induced alterations in cellular and molecular components of the blood–brain barrier. Accurate measurement of BBB total leakage can provide valuable insights into disease progression that might support clinical decision-making. Although previous studies have reported disease-specific changes in heterogeneous components of the BBB,^6^^,^^20^^,^^34^^,^^58^ corresponding changes in BBB total leakage itself have not been fully characterized yet. For treating increased BBB total leakage under a disease state,^12^^,^^16^ it is necessary to clarify which BBB components are predominantly altered in each disease and to accurately understand how these changes affect the alteration in BBB total leakage. Furthermore, this approach has potential applications in brain drug delivery, allowing real-time observation of drug transport across the BBB and facilitating a deeper understanding of delivery mechanisms.

The method presented here, which combines *in vivo* two-photon live imaging with UMAP analysis, offers a more sensitive and reliable means of identifying differences in extravasation patterns. To expand and use the proposed method in the future, it is necessary to consider the following variables. The reason for designating the perivascular area in the range of 5 to 15  μm from the blood vessel to be used for the extraction of a single-vessel extravasation was to avoid signals from the corresponding vessel and adjacent vessels.^44^ The reason for designating the perivascular area in the range of 5 to 15  μm from the blood vessel for the extraction of single-vessel extravasation was to minimize direct signals from the targeted vessel and to reduce contamination from adjacent vessels. However, given the dense vascular architecture of the brain, in which most parenchymal cells lie within 10 to 15  μm of at least one capillary, we acknowledge that signals measured within this ROI may still be influenced by nearby microvessels, including small arterioles or venules. In cases where perivascular ROIs overlapped, the shared parenchymal signal was assigned to the nearest vessel. Although this approach reduces—but does not completely eliminate—signals from other vessels, it provides a consistent framework for isolating vessel-specific extravasation. This methodological limitation has been considered in the interpretation of our findings. Extravasated tracers have different diffusion rates depending on molecular weight or size.^59^^–^^61^ To apply the proposed method to various tracers, it is necessary to optimize the perivascular area accordingly. Furthermore, to optimize tracer visualization, *in vivo* two-photon imaging parameters were adjusted to achieve optimal spatial and temporal resolution. Subsequently, single-vessel extravasation patterns were analyzed using UMAP, a dimensionality reduction technique, to derive a BBB total leakage index. The extracted UMAP distance demonstrated higher sensitivity and reliability than AUC, $\Delta F/F_{0}$, and ΔF/Δt. It also allowed for visualization of single-vessel extravasation patterns in both disease and control groups. Extravasation patterns may vary across different disease states. To extend the applicability of this approach to diverse pathological conditions, tracer-specific hyperparameter optimization is essential. Furthermore, given that diverse dimensionality reduction techniques have recently emerged, alternative methods could also be explored.^52^^,^^62^^,^^63^ In addition, to assess the diversity of a specific disease, cluster distribution within the disease needs to be distinguished by various clustering methods.^28^^,^^52^^,^^63^ In summary, this study introduces a novel single-vessel extravasation pattern analysis that can reveal heterogeneous BBB changes based on specific disease state and vascular type.

## 5 Conclusion

This study introduces a single-vessel extravasation pattern analysis that leverages *in vivo* two-photon live imaging with dimensionality reduction to quantitatively assess heterogeneous BBB total leakage. Through this method, we demonstrated that even among diseases known to increase BBB total leakage (SE vs. NI), distinct changes in BBB components, could further influence the total leakage of specific blood vessel types. As such, the proposed method holds promise for quantifying transcytosis, characterizing BBB total leakage in diverse disease models.

## Supplementary Material

10.1117/1.NPh.12.4.045004.s01

## Data Availability

The experimental data and custom analysis code used in this study are available from the corresponding author upon reasonable request.
