# Supplementary material for: Real-time single-vessel analysis reveals vascular-type-dependent blood–brain barrier dysfunction in rodent models of status epilepticus and neuroinflammation
Source: Neurophotonics. 2025 Oct 22;12(4):045004. doi: 10.1117/1.NPh.12.4.045004 (PMC12543165; doi:10.1117/1.NPh.12.4.045004)
Supplement: Supplementary file 1 [file NPh_012_045004_SD001.pdf]

Supplementary Material

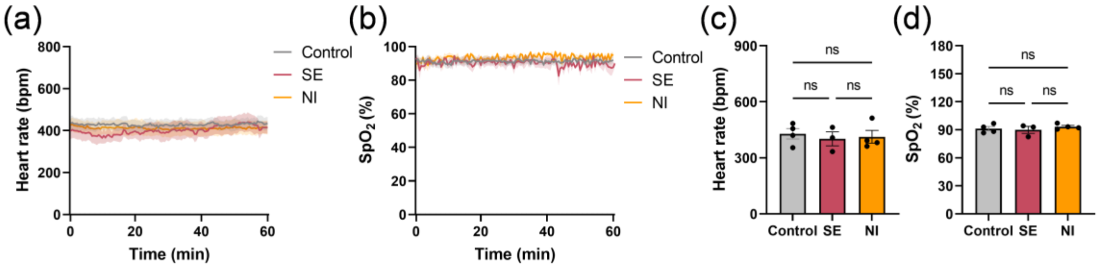

Fig. S1 Heart rate and blood oxygen saturation (SpO_2) of animals during imaging sessions. (a) Heart rate traces over time. (b) SpO_2 traces over time. (c) Mean heart rate. (d) Mean SpO_2. Control, n=4; SE, n=3; NI, n=4. Data are presented as mean ± SEM. Kruskal-Wallis test with Bonferroni-corrected post-hoc analyses was used for multiple comparisons. ns, not significant.

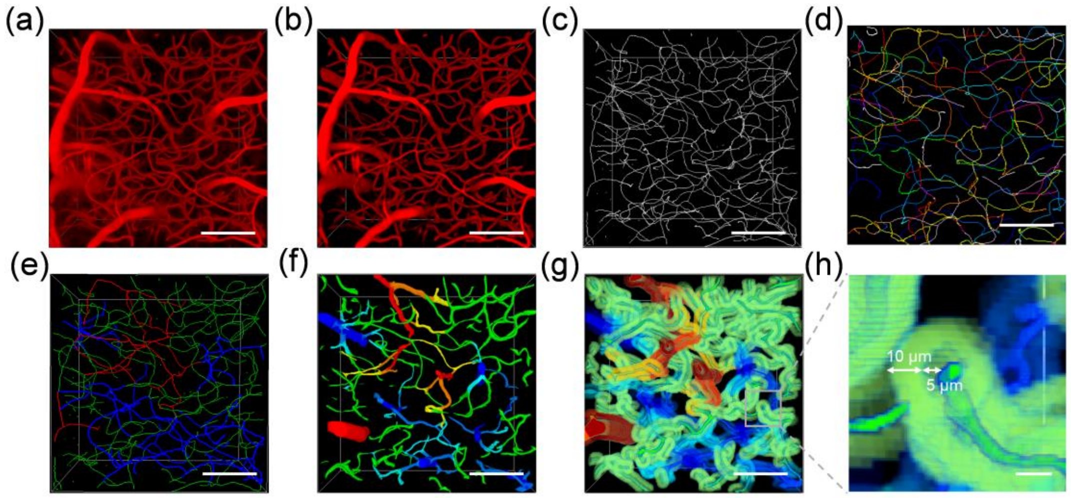

Fig. S2 Processing steps for determining vascular types and perivascular regions. (a) Raw image. (b) Image preprocessed and binarized using Gaussian filtering, background correction, and z-normalization. (c) Skeletonized image generated from the binary image. (d) Network graph structure calculated from the skeletonized image, with colors indicating individual vessels. (e) Manually classified arterial vessels, microvessels, and venous vessels. (f) and (h) Reconstructed arterial vessels, microvessels, and venous vessels along with their corresponding perivascular regions. Color code: Red, 0th-order arteriole; Orange, 1st-order arteriole; Yellow, 2nd-order arteriole; Green, capillary; Cyan, 2nd-order venule; Sky blue, 1st-order venule; Blue, 0th-order venule. Scale bars: 100 μm for original image; 10 μm for enlarged image.

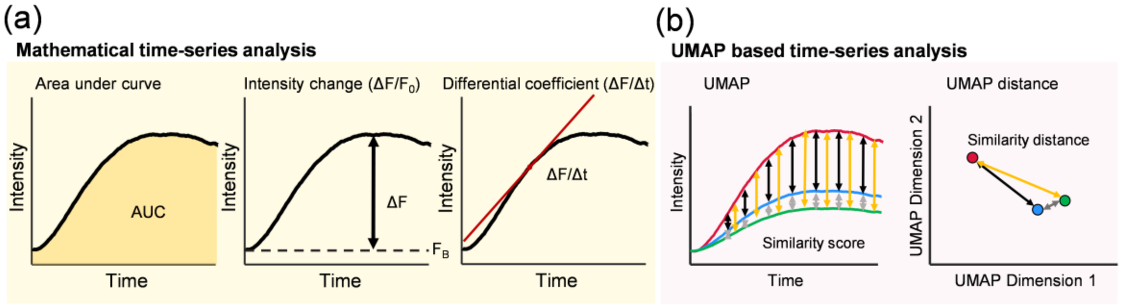

Fig. S3 *In vivo* time-series analysis of perivascular extravasation. (a) Mathematical time-series analysis. AUC (pixel intensity over time), intensity change ($\Delta F/F_0$), and an averaged differential coefficient ($\Delta F/\Delta t$). (b) UMAP-based time-series analysis.

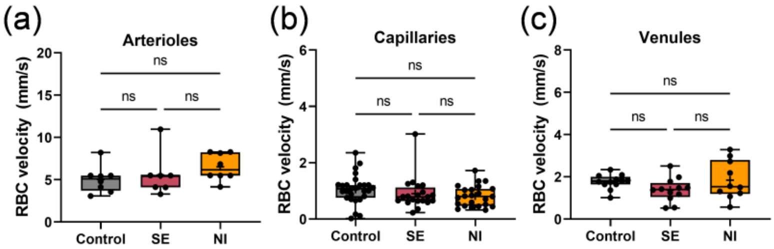

Fig. S4 RBC velocity measurement by vascular type in control, SE and NI groups. (a-c) RBC velocity for each vessel type analyzed in the main experiments (arteriole, capillary, venule) under control, SE, and NI conditions. Control, n = 4 (8 arterioles, 27 capillaries, 12 venules); SE, n = 3 (7 arterioles, 20 capillaries, 10 venules); NI, n = 4 (8 arterioles, 23 capillaries, 12 venules). Dots are individual vessel segments. Median values are indicated by black lines, and mean values by “+” symbols. Kruskal-Wallis test with Bonferroni-corrected post-hoc analyses was used for multiple comparisons. ns, not significant.

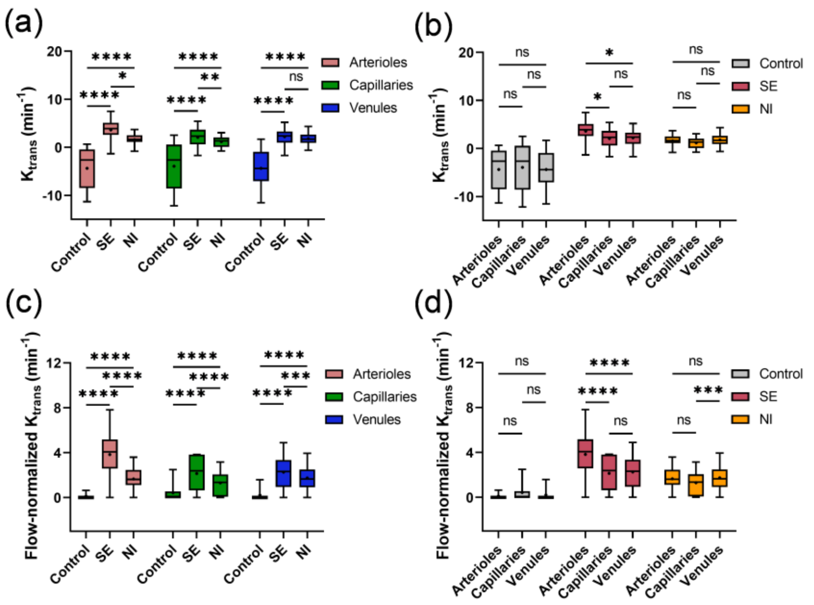

Fig. S5 Vessel-type–dependent BBB permeability calculated by both the volume transfer constant (K_{trans}) and a flow-normalized K_{trans}. (a-b) Volume transfer constant (K_{trans}). (c-d) Flow-normalized K_{trans}. Control, n = 4 (39 arterioles, 383 capillaries, 193 venules); SE, n = 4 (47 arterioles, 323 capillaries, 169 venules); NI, n = 3 (44 arterioles, 285 capillaries, 168 venules). Median values are indicated by black lines, and mean values by "+" symbols. One-way ANOVA followed by Bonferroni-corrected post-hoc analyses was used for multiple comparisons. *, $p < 0.05$; **, $p < 0.01$; ***, $p < 0.001$; ****, $p < 0.0001$; ns, not significant.

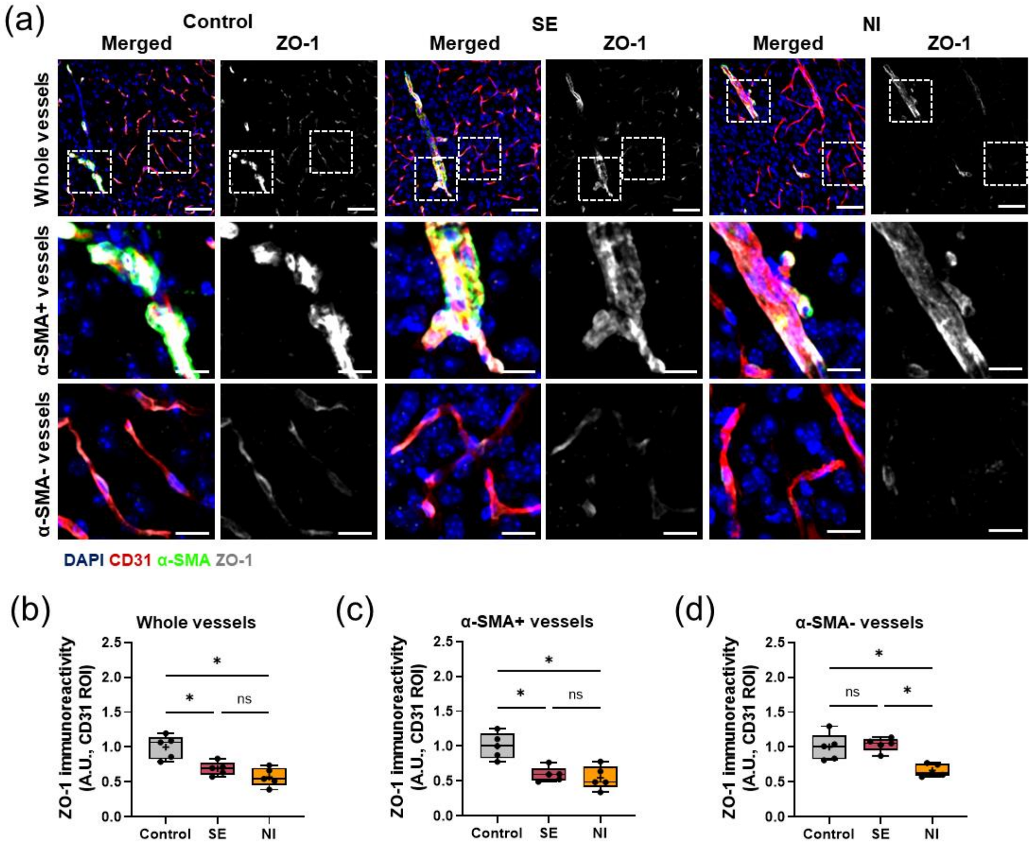

Fig. S6 Loss of tight-junction protein ZO-1 in disease models. (a) Representative confocal images for Control, SE, and NI showing merged channels (DAPI, CD31, α-SMA, ZO-1) and the ZO-1 channel alone. Rows display whole vessels (CD31+), α-SMA+ vessels, and α-SMA- vessels. Scale bars: 50 μm for original image; 15 μm for enlarged image. (b-d) Quantification of ZO-1 expression within CD31-defined vessel ROIs. (b) Whole vessels. (c) α-SMA+ vessels. (d) α-SMA- vessels. Control, n = 5; SE, n = 5; NI, n = 5. Median values are indicated by black lines, and mean values by "+" symbols. Kruskal-Wallis test with Bonferroni-corrected post-hoc analyses was used for multiple comparisons. *, $p < 0.05$; ns, not significant.

Table S1 Descriptive statistics of blood-brain barrier (BBB) leakage indices

Vascular type		Arterioles		Capillaries		Venules		Total	
Control	Number	39		383		193		615	
		Averaged	SD.	Averaged	SD.	Averaged	SD.	Averaged	SD.
	UMAP	1.000	0.074	1.000	0.087	1.000	0.096	1.000	0.085
	AUC	64.230	10.720	66.062	12.753	63.584	11.364	65.189	11.996
	$\Delta F/F_0$	1.000	0.271	1.000	0.292	1.000	0.264	1.000	0.277
	$\Delta F/\Delta t$	-0.002	0.020	0.002	0.023	-0.002	0.020	0.000	0.022
SE	Number	47		323		169		539	
		Averaged	SD.	Averaged	SD.	Averaged	SD.	Averaged	SD.
	UMAP	1.734	0.230	1.428	0.155	1.334	0.095	1.415	0.139
	AUC	84.802	8.689	82.361	5.221	80.735	2.484	81.982	4.582
	$\Delta F/F_0$	1.438	0.309	1.334	0.166	1.360	0.109	1.348	0.158
	$\Delta F/\Delta t$	0.034	0.026	0.029	0.014	0.026	0.009	0.028	0.013
NI	Number	44		285		168		497	
		Averaged	SD.	Averaged	SD.	Averaged	SD.	Averaged	SD.
	UMAP	1.296	0.345	1.319	0.112	1.422	0.091	1.404	0.078
	AUC	77.765	6.821	80.923	1.342	82.699	3.800	82.319	3.619
	$\Delta F/F_0$	1.280	0.115	1.315	0.081	1.421	0.124	1.365	0.109
	$\Delta F/\Delta t$	0.021	0.008	0.027	0.011	0.030	0.013	0.030	0.012

SE, status epilepticus model; NI, neuroinflammation model; UMAP, uniform manifold approximation and projection; AUC, area under curve; $\Delta F/F_0$, intensity fold change; $\Delta F/\Delta t$, averaged differential coefficient

Table S2 Inferential statistics of BBB leakage indices

	Comparison	UMAP	AUC	$\Delta F/F_0$	$\Delta F/\Delta t$
**Arterioles**	**Control vs. SE**	< 0.001	< 0.001	< 0.001	< 0.001
	**Control vs. NI**	< 0.001	< 0.001	< 0.001	< 0.001
	**SE vs. NI**	< 0.001	< 0.001	< 0.001	< 0.001
**Capillaries**	**Control vs. SE**	< 0.001	< 0.001	< 0.001	< 0.001
	**Control vs. NI**	< 0.001	< 0.001	< 0.001	< 0.001
	**SE vs. NI**	0.726	0.290	0.321	0.464
**Venules**	**Control vs. SE**	< 0.001	< 0.001	< 0.001	< 0.001
	**Control vs. NI**	< 0.001	< 0.001	< 0.001	< 0.001
	**SE vs. NI**	0.003	0.054	0.654	0.642
**Total**	**Control vs. SE**	< 0.001	< 0.001	< 0.001	< 0.001
	**Control vs. NI**	< 0.001	< 0.001	< 0.001	< 0.001
	**SE vs. NI**	0.157	0.669	.214	.082

Sample size: Arterioles (Control, 39 vessels; SE, 47 vessels; NI, 44 vessels), Capillaries (Control, 383 vessels; SE, 323 vessels; NI, 285 vessels), Venules (Control, 193 vessels; SE, 169 vessels; NI, 168 vessels), Total (Control, 615 vessels; SE, 539 vessels; NI, 497 vessels)
